# Astaxanthin alleviates oxidative stress and skeletal muscle damage by promoting mitochondrial biogenesis

**DOI:** 10.3389/fvets.2025.1577408

**Published:** 2025-09-02

**Authors:** Chengmu Li, Yining Yan, Kai Wang, Tao Jiang

**Affiliations:** 1 Department of Orthopaedics, The Second Affiliated Hospital of Army Military Medical University, Chongqing, China; 2 Jilin Univ, Dept Radiol, China Japan Union Hosp, Xi’an, China; 3 Department of Spine Surgery, Honghui Hospital, Xi’an Jiaotong University, Xi’an, China

**Keywords:** astaxanthin, skeletal muscle, mitochondrial biogenesis, oxidative stress, high-fat diet

## Abstract

**Introduction:**

This study aimed to investigate the damaging effects of a high-fat diet (HFD) on mitochondria and skeletal muscle and to evaluate the protective role of astaxanthin (Asta), with a focus on mitochondrial biogenesis, oxidative stress, and inflammation under metabolic stress.

**Methods:**

HFD-fed mice and palmitate acid (PA)-stimulated C2C12 cells were treated with Asta. Skeletal muscle function, pathology, mitochondrial ultrastructure, inflammatory responses, and oxidative stress levels were assessed using behavioral tests, histology, quantitative reverse transcription-polymerase chain reaction, western blotting, transmission electron microscopy, and biochemical assays.

**Results:**

Asta did not alter body weight or serum lipid levels in HFD-fed mice but markedly alleviated skeletal muscle damage and improved function. In both *in vivo* and *in vitro* models, Asta suppressed inflammatory gene expression, enhanced mitochondrial biogenesis-related proteins, reduced lipid accumulation and mitochondrial damage, increased antioxidant enzyme activity, and promoted ATP production. Furthermore, Asta inhibited mitochondrial fission and lipid peroxidation in PA-stimulated C2C12 cells.

**Discussion:**

Asta mitigates oxidative stress, lipid accumulation, and inflammation in skeletal muscle cells by promoting mitochondrial biogenesis, thereby preserving muscle structure and function. These findings highlight Asta’s potential as a therapeutic agent for skeletal muscle protection in metabolic stress conditions.

## Introduction

Skeletal muscle plays a pivotal role in regulating metabolism, physical performance, and overall health. However, in the context of a high-fat diet (HFD), skeletal muscle is one of the primary tissues that suffer from metabolic dysfunction (1). Excessive lipid accumulation within muscle fiber leads to insulin resistance, impaired glucose uptake, and inflammation reaction, which are key contributors to metabolic diseases such as obesity and type 2 diabetes mellitus (T2DM) (2, 3). In addition to these metabolic alterations, HFD results in structural and functional damage to skeletal muscle cells (4).

Mitochondria are essential for energy production and metabolic homeostasis. In skeletal muscle, mitochondrial function is extremely critical for maintaining muscle endurance and strength. However, HFD disrupts mitochondrial dynamics, leading to decreased mitochondrial biogenesis, fragmentation of existing mitochondria, and impaired oxidative phosphorylation ability (1, 5). Furthermore, an excess lipid deposition leads to the overproduction of reactive oxygen species (ROS), which subsequently exacerbates oxidative damage to mitochondrial membranes, proteins, as well as DNA (2, 6, 7). These mitochondrial dysfunctions not only impair energy production but also accelerates skeletal muscle atrophy and reduces muscle performance (4, 5, 8). Given the central role of mitochondria in skeletal muscle deterioration, improving mitochondrial function is a promising therapeutic approach. Studies have demonstrated that enhancing mitochondrial biogenesis can alleviate its damage, restore energy metabolism, reduce oxidative stress, and protect against muscle injury (5, 9, 10). Currently, therapeutic interventions that target mitochondrial homeostasis have shown potential in mitigating muscle damage and improving overall muscle health.

Astaxanthin (Asta), a potent antioxidant derived from marine organisms, has been studied for its protective effects on various tissues, particularly in conditions of oxidative stress and inflammation (11–13). In the brain tissue, Asta has been reported to reduce neuroinflammation and protect against neurodegenerative diseases (13). In the cardiovascular tissues, it has demonstrated the ability to reduce oxidative stress, enhance endothelial function, and protect against atherosclerosis (14, 15). Furthermore, the effects of Asta on liver protection in non-alcoholic fatty liver disease models highlight its potential for managing metabolic stress (16, 17). Despite these well-documented protective effects in various tissues, the role of Asta in skeletal muscle, especially under the stress of HFD, remains inadequately explored.

Our study aimed to investigate the effects of Asta on mitochondria and skeletal muscle in HFD-fed mice. Here, we hypothesize that Asta treatment could alleviate skeletal muscle damage by enhancing mitochondrial biogenesis and reducing oxidative stress. In this study, we treated HFD-fed mice and palmitic acid (PA)-exposed C2C12 cells with Asta and assessed changes in mitochondrial biogenesis, oxidative stress, and skeletal muscle cells.

## Materials and methods

### Animals study and administration

**Figure 1:**
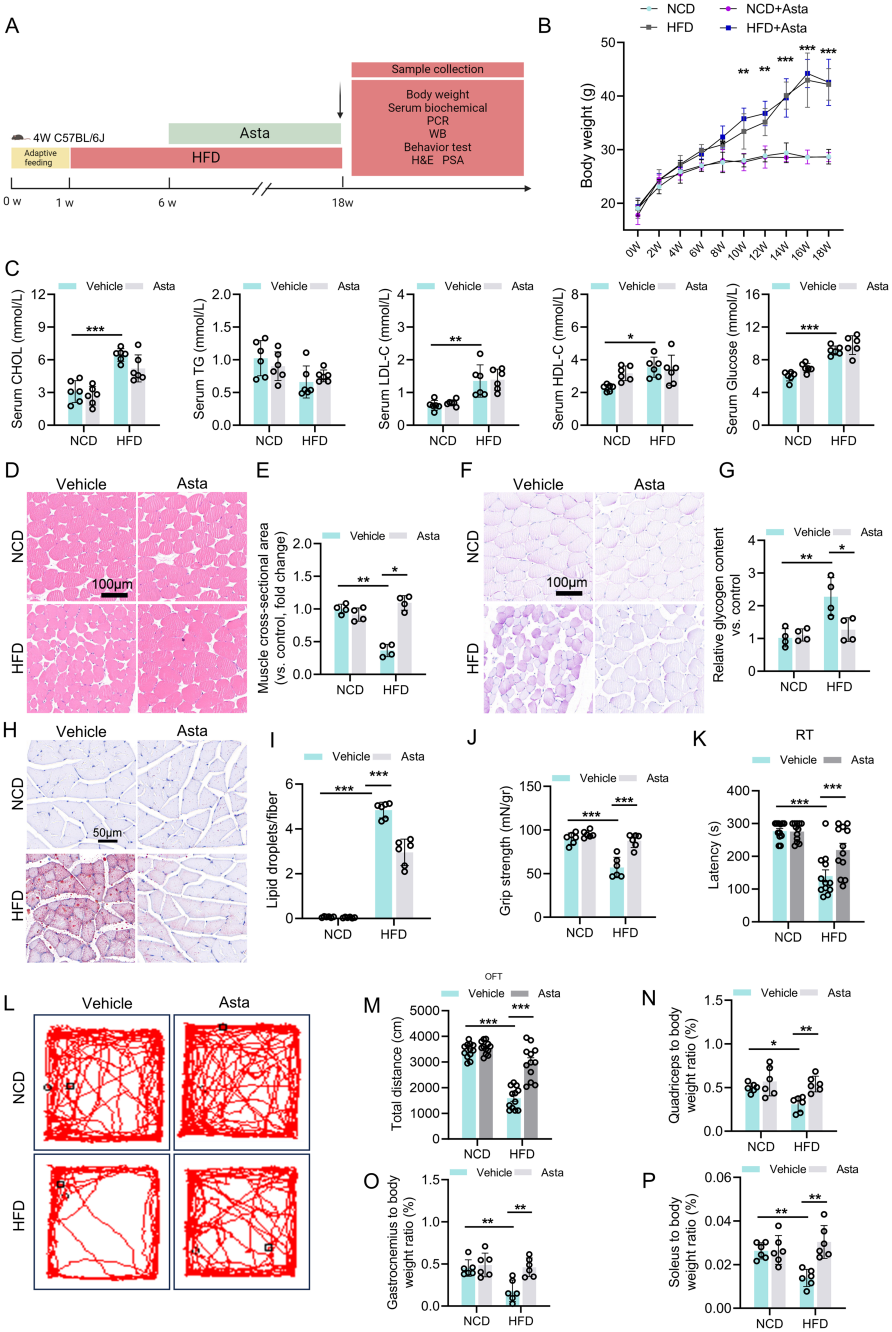
Astaxanthin (Asta) treatment alleviates HFD-induced skeletal muscle damage in mice. **(A)** A representative image of experimental design: Mice were fed a normal chow diet (NCD) or high-fat diet (HFD) for 18 weeks, followed by vehicle or Asta treatment (200 mg/kg) for the final 12 weeks. **(B,C)** Body weight, serum cholesterol (CHOL), triglycerides (TG), low-density lipoprotein cholesterol (LDL-C), high-density lipoprotein cholesterol (HDL-C), and glucose levels (*n* = 6). **(D–G)** Pathological staining (H&E and PAS) and quantitative analysis (*n* = 4). **(H,I)** Oil Red staining of frozen sections of skeletal muscle and quantitative analysis (*n* = 4). **(J)** Evaluation of grip strength in muscles of mice (*n* = 6). **(K)** Behavioral of rotarod test (RT) of mice (*n* = 12). **(L,M)** Open filed test of mice. Representative tracking images **(L)** and total distance of mice **(M)** (*n* = 12). **(N–P)** Muscle mass-to-body weight ratio (*n* = 6). Data are shown as mean ± standard deviation (SD). Statistical analysis among four groups was performed using two-way ANOVA with a Sidak *post hoc test*. * *p* < 0.05, ** *p* < 0.01, and *** *p* < 0.001.

Male C57BL/6 J mice (4 weeks old) were purchased from Beijing Vital River Laboratory Animal Technology Co. Ltd. Mice were housed under standard conditions (22 ± 2 °C, 12 h light/dark cycle) with free access to food and water. After 1 week of acclimatization, mice were randomly assigned to either a normal chow diet (NCD) group or HFD (D12492, containing 60% fat, Research Diets, New Brunswick, United States) group. Following 6 weeks of the dietary intervention, HFD-fed mice were further randomized to receive either the vehicle (hydroxypropyl-*β*-cyclodextrin, *n* = 6) or Asta (HY-B2163, MedChemExpress, United States; purity ≥ 98%) at a dose of 200 mg/kg via oral gavage every other day for 12 weeks. Similarly, NCD-fed mice received the same vehicle (hydroxypropyl-*β*-cyclodextrin, *n* = 6) or Asta (200 mg/kg, *n* = 6) for the same duration. The overall experimental timeline is illustrated in Figure 1A. At the end of the 18-week feeding period, mice were anesthetized using isoflurane (induction: 3.0–4.0%, maintenance: 1.5–2.0%; RWD Life Science, China) in 100% oxygen (1.0 L/min) via a nose cone. Blood samples were collected from the retro-orbital sinus under deep anesthesia. Mice were subsequently euthanized by cervical dislocation, and quadriceps muscles were immediately harvested for subsequent experiments. All experimental procedures were conducted in accordance with the National Institutes of Health Guide for the Care and Use of Laboratory Animals (NIH Publication No. 8023, revised 1978) and were approved by the institutional animal care and use committee.

### Grip strength test

To assess muscle strength, a grip strength meter (BioSeb, France) was used to measure the forelimb grip strength of the mice. The test was performed at the end of the 18-week treatment period. Before the actual measurements, mice were acclimated to the device for two consecutive days to minimize stress and ensure consistency in the results. For the test, each mouse was gently lifted by the tail and allowed to grasp a horizontal metal bar attached to the grip strength meter with its forelimbs. Once the mouse had a firm grip, it was pulled backward steadily until it released the bar. The force applied by the mouse before releasing the bar was recorded in grams. Each mouse was tested three times, and the average grip strength was calculated. The grip strength data were normalized to the body weight of each mouse to account for differences in body size. The normalized values (grip strength/body weight) were used for statistical analysis to evaluate muscle function across the different treatment groups.

### Rotarod test

Motor coordination was evaluated using a rotating rod (6 cm diameter, coarse surface) designed for optimal grip. Mice were habituated to the testing environment for 2 h before testing. For training, animals were placed on the rod with the speed increasing from 5 to 10 rpm during a 30 min session. On the test day, the rotation accelerated from 5 to 40 rpm over 5 min. The latency to fall was automatically recorded for each trial. Four trials were conducted per mouse per day, with 30 min intervals between trials. The apparatus was cleaned with 75% ethanol after each animal.

### Open field test

The OFT was performed as previously described, with minor modifications. Briefly, mice were individually placed in the center of a square open field arena (40 × 40 × 40 cm, white Plexiglas) and allowed to explore freely for 10 min. Locomotor activity was recorded using an overhead video camera and analyzed with automated tracking software (Shanghai Xinruan Information Technology Co., Ltd.). The total distance traveled and time spent in the central zone (20 × 20 cm) were quantified. The apparatus was cleaned with 70% ethanol between trials to eliminate olfactory cues. All behavioral assays were conducted during the light phase (09:00–17:00) under consistent illumination (100 lux).

### Serum lipid profile measurement

At the end of the treatment period, blood samples were collected from the retro-orbital sinus under anesthesia. Then, blood of mice was centrifugated at 3,000 rpm for 15 min at room temperature to obtain serum. The assay kits were purchased from Nanjing Jiancheng Bioengineering Institute (Jiangsu, China) and were used to detect lipid and glucose profiles. Serum total cholesterol (CHOL, A111-1-1), triglycerides (TG, A110-1-1), glucose (A154-1-1), low-density lipoprotein cholesterol (HDL-C, A112-1-1), and high-density lipoprotein cholesterol (LDL-C, A113-1-1) levels were tested with a microplate reader (Multiskan SkyHigh, Thermo Fisher Scientific, United States).

### Muscle mass and muscle-to-body weight ratio

The quadriceps, gastrocnemius, and soleus muscles were harvested from mice and weighed. The muscle mass was normalized to the body weight of each mouse to calculate the muscle-to-body weight ratio. This ratio was used as an important indicator of muscle atrophy or hypertrophy.

### Histological evaluation of quadriceps muscle

At the end of the 12-week treatment, mice were euthanized, and quadriceps muscles were excised, fixed with 4% paraformaldehyde, and embedded in paraffin. Tissue sections (5 μm) were stained with hematoxylin and eosin (H&E) and periodic acid-schiff (PAS) to assess mouse muscle morphology in accordance with standard procedures (MXB, Fujian, China). Images were then captured with a light microscope (Leica). Muscle fiber cross-sectional area was measured on H&E-stained sections. Five randomly selected non-overlapping fields per section were analyzed. Using ImageJ software, at least 100 muscle fibers with clear boundaries were manually outlined per sample, and their areas were then calculated. Glycogen content was performed using ImageJ software.

### Transmission electron microscopy of skeletal muscle mitochondria

Freshly dissected skeletal muscle tissues were immediately fixed in 2.5% glutaraldehyde in 0.1 M phosphate buffer (pH 7.4) at 4 °C to preserve ultrastructure. After rinsing in phosphate buffer, samples were embedded in 1% low-melting-point agarose for orientation. Post-fixation was performed with 1% osmium tetroxide at 4 °C to enhance membrane contrast. Tissues were then dehydrated through a graded acetone series, infiltrated, and embedded in epoxy resin. Ultrathin sections (60–80 nm) were cut using an ultramicrotome, collected on copper grids, and stained sequentially with uranyl acetate and lead citrate. Sections were imaged with a transmission electron microscope (e.g., Hitachi HT7800) operated at 80 kV. Images were acquired digitally for quantitative assessment of mitochondrial morphology.

### Skeletal muscle cells culture

C2C12 murine skeletal muscle cells were (China Infrastructure of Cell Line Resource, Beijing) cultured in high-glucose Dulbecco’s modified Eagle’s medium (DMEM, Gibco, China) supplemented with 10% fetal bovine serum (FBS) and 1% penicillin–streptomycin at 37 °C with 5% CO_2_. Cells were differentiated into myotubes by switching to DMEM medium containing 2% horse serum for 5 days. After differentiation, C2C12 cells were treated with PA (0–300 μM) to mimic high-fat conditions *in vitro* for 36 h, followed by the vehicle (0.1% DMSO) or Asta treatment (150 μM) during the last 24 h. After treatment, C2C12 cells were harvested for analysis of cell activity, mitochondrial morphology, and mitochondrial biogenesis and oxidative stress.

### Oil Red O staining

C2C12 myotubes were fixed in 4% paraformaldehyde for 20 min, while skeletal muscle sections (8–10 μm) were air-dried and fixed similarly for 15 min. After PBS washing, samples were incubated with freshly prepared ORO working solution for 15–20 min at room temperature, then rinsed with distilled water. Tissue sections were counterstained with hematoxylin. Lipid droplets were visualized and imaged by light microscopy. For quantification in cells, the dye was eluted with isopropanol and absorbance measured at 500 nm.

### Cellular TG measurement

Intracellular TG levels in C2C12 cells were measured using a commercial assay kit (A110-1-1, Nanjing Jiancheng Bioengineering Institute, Nanjing, China) according to the standard instructions. Briefly, after treatment, cells were washed twice with cold PBS and lysed in the provided lysis buffer. The lysates were collected and centrifuged at 12,000 rpm for 10 min at 4 °C. The supernatant was used to determine TG content based on enzymatic colorimetric reaction, and absorbance was measured at 510 nm using a microplate reader (Multiskan SkyHigh, Thermo Fisher Scientific, United States). Protein concentration was determined using a BCA assay, and TG levels were normalized to total protein content (nmol/mg protein).

### Cell viability assay

C2C12 myoblasts were seeded in 96-well plates (5,000 cells/well) and then differentiated into mature myotubes by incubation in DMEM medium with 2% horse serum for 5 days. Cells were next treated with PA and Asta. Cell viability was assessed using CCK-8 assay kit (Dojindo, Japan) by adding 10 μL of reagent to each well and incubating for 2 h at 37 °C. Absorbance was measured at 450 nm, and cell viability was expressed as a percentage of the control group. All experiments were conducted in triplicate.

### Quantitative reverse transcription-polymerase chain reaction

Total RNA was extracted from mouse skeletal muscle tissues (quadriceps) and C2C12 cells using TRIzol reagent (Invitrogen, United States) following manufacturer’s protocol. RNA concentration and purity were determined using a NanoDrop spectrophotometer (Thermo Fisher). One microgram of RNA was reverse-transcribed into cDNA using a PrimeScript RT reagent kit (Takara, Japan). Quantitative reverse transcription-polymerase chain reaction (qRT-PCR) was performed using SYBR Green Master Mix (Yeasen Biotechnology, Shanghai, China) on an ABI 7500 RT-PCR system. The expression levels of target genes (mitochondrial quality network, inflammation, and oxidative stress) were quantified and normalized to the housekeeping gene of *Gapdh*. The relative expression levels were calculated using the 2^−ΔΔCt^ method. The primers were obtained from Shanghai Sangon Biotechnology (China) and are listed in Supplementary Table S1.

### Western blotting analysis

Total protein was extracted from mouse skeletal muscle tissues (quadriceps) and C2C12 cells using RIPA lysis buffer (Beyotime, Shanghai, China) supplemented with protease inhibitors. Protein concentrations were determined using a BCA Protein Assay Kit (Beyotime). Equal amounts of protein (30 μg) from each sample were separated by SDS-PAGE and then transferred onto PVDF membranes (Millipore, United States). The blots were blocked with 5% non-fat milk mixed in tris-buffered saline with 0.1% tween-20 (TBST) for 1 h at 37 °C. Subsequently, the blots were incubated with primary antibodies against peroxisome proliferator-activated receptor gamma coactivator 1-alpha (PGC-1α, Proteintech Group, 66,369-1-Ig, 1:5000), transcription factor A, mitochondrial (TFAM, Proteintech Group, 22,586-1-AP, 1:2000), mitochondrial F1 complex, alpha subunit 1 (ATP5A, Proteintech Group, 66,037-1-Ig, 1:5000), and glyceraldehyde −3-phosphate dehydrogenase (GAPDH, Signalway Antibody, SAB, United States, #21612, 1:7500) overnight at 4 °C, next followed by an incubation of horseradish peroxidase (HRP)-conjugated secondary antibodies (Abcam, a–b6721, 1:15000; Sigma-Aldrich, A9044, 1:80000) for 1 h at room temperature. After washing with TBST, blots were visualized using chemiluminescence (ECL) detection kit (ECL, E411-05, Vazyme Biotech, Nanjing, China) and detected using Multi Tanon 5,200 imaging system. The protein expression was quantified by densitometry using ImageJ software.

### Superoxide dismutase and glutathione peroxidase activity assays

Skeletal muscle tissues (quadriceps) and C2C12 cells were homogenized in ice-cold phosphate-buffered saline (PBS, pH = 7.20–7.40) and centrifuged at 12,000 rpm for 10 min at 4 °C. Obtained supernatants were collected for further analysis. SOD activity assay kit (Sigma-Aldrich, CS0009, St. Louis, MO, United States) was used to measure SOD activity in muscle homogenates. GPx assay kit (Abcam, ab102530, Cambridge, United Kingdom) was used for the quantification of the activity of glutathione-dependent peroxidases as a marker of antioxidant activity. SOD activity was expressed as units per milligram of protein (U/mL), while GPx activity was expressed as nanomoles of NADPH consumed per minute per milligram of protein (U/pg. protein).

### MDA and ATP assay

A Lipid Peroxidation Assay Kit (Abcam, ab118970, Cambridge, United Kingdom) was used for colorimetric detection of the lipid peroxidation marker malondialdehyde (MDA) in quadriceps muscle and C2C12 cells homogenates by a reaction with thiobarbituric acid reactive substances (TBARS). All samples, controls, and standards were assayed in duplicate. The optical density (OD) of wells was determined using a Varioskan LUX Multimode Microplate Reader set to 532 nm. MDA levels were expressed as nanomoles per milligram of protein (nmol/mg protein). ATP levels in muscle tissues and C2C12 cells were measured using corresponding commercial kit (Jiancheng Institute of Bioengineering, Nanjing, China) according to manufacturer’s instructions.

### Mitochondrial staining in C2C12 cells

C2C12 cells were seeded on coverslips and then differentiated into myotubes for 5 days. After treatment, cells were incubated with Mito-Tracker Red FM (100 nM, Thermo Fisher) for 30 min at 37 °C. Cells were washed with PBS, fixed with 4% paraformaldehyde for 15 min, and mounted with DAPI-containing medium. Fluorescence images were captured using a confocal microscope, and mitochondrial morphology was analyzed using ImageJ.

### Lipid peroxidation assay in C2C12 cells

C2C12 myotubes were treated with PA for 36 h, followed by Asta treatment. Lipid peroxidation was assessed using BODIPY™ 581/591 C11 (Beyotime, Shanghai, China) by incubating cells at 37 °C for 30 min. After washing with PBS, C2C12 cells were imaged using a fluorescence microscope. The green/red fluorescence ratio, indicating lipid peroxidation, was quantified using ImageJ software.

### TNF-*α* and IL-1β assessment in C2C12 cells

After treatment with 300 μM PA for 36 h and last 24-h Asta treatment, C2C12 cells were lysed in RIPA buffer containing protease inhibitors. The cell lysates were centrifuged at 12,000 rpm for 10 min at 4 °C, and the supernatants were collected. TNF-α and IL-1β protein levels were quantified using enzyme linked immunosorbent assay (ELISA) kits (MULTI SCIENCES, China) according to manufacturer’s protocols. The optical density (OD) was measured at 450 nm using a microplate reader. Results were normalized to total protein content as determined by the BCA protein assay.

### Statistical analysis

All data are presented as mean ± standard deviation (SD). Statistical significance between groups was evaluated using two-way analysis of variance (ANOVA) followed by a Sidak multiple comparison *post hoc test*. A *p*-value <0.05 was considered statistically significant. Data analysis was performed using GraphPad Prism software (version 10.0).

## Results

### Asta treatment alleviates HFD-induced skeletal muscle damage in mice

We first explore the role of Asta in the skeletal muscle of mice (Figure 1A). Mice fed a HFD for 18 weeks exhibited significant increase in body weight, serum CHOL, LDL-C, HDL-C, and glucose levels compared to those fed NCD (Figures 1B,C). These changes indicate that HFD feeding for 18 weeks induced metabolic disruption, as is observed in models established obesity. Treatment with Asta for 12 weeks was not effective in reducing body weight, serum lipid, and glucose levels in HFD-fed mice (Figures 1B,C), suggesting that Asta did not directly influence overall body weight or systemic lipid/glucose metabolism under this condition.

Histological analysis of quadriceps muscles revealed that HFD feeding caused structural changes in the skeletal muscle fibers. Specifically, cross-sectional area of the muscle fibers in HFD-fed mice was significantly reduced compared to that of the NCD group (Figures 1D,E), which indicated muscle atrophy and fiber thinning as a result of HFD feeding. PAS staining showed a significant increase in glycogen accumulation in the quadriceps muscles of HFD-fed mice (Figures 1F,G), reflecting altered glucose metabolism and storage within the muscle tissues. Asta treatment for 12 weeks resulted in a marked reversal of these alterations (Figures 1D–G). The results of ORO staining exhibited significant lipid accumulation in the skeletal muscle of HFD-fed mice, which was markedly decreased by Asta treatment (Figures 1H,I).

Assessment of muscle strength demonstrated that HFD-fed mice exhibited reduced grip strength relative to NCD controls, whereas Asta supplementation significantly improved muscle strength in the HFD group (Figure 1J). Motor coordination and endurance, as measured by RT, were also impaired in HFD-fed mice but substantially improved upon Asta administration (Figure 1K). OFT analysis showed that HFD-fed mice exhibited decreased locomotor activity, as evidenced by a reduction in total distance traveled (Figures 1L,M). Notably, Asta treatment rescued locomotor deficits in the HFD group. Muscle mass assessments further revealed that HFD significantly increased the ratios of quadriceps (Figure 1N), gastrocnemius (Figure 1O), and soleus (Figure 1P) to body weight, which were partially normalized by Asta supplementation. Collectively, these findings underscore the potential protection of Asta in muscle function and lass, even in the presence of metabolic disturbances.

### Asta treatment alleviates HFD-induced oxidative stress and impaired mitochondrial biogenesis in skeletal muscles of mice

**Figure 2:**
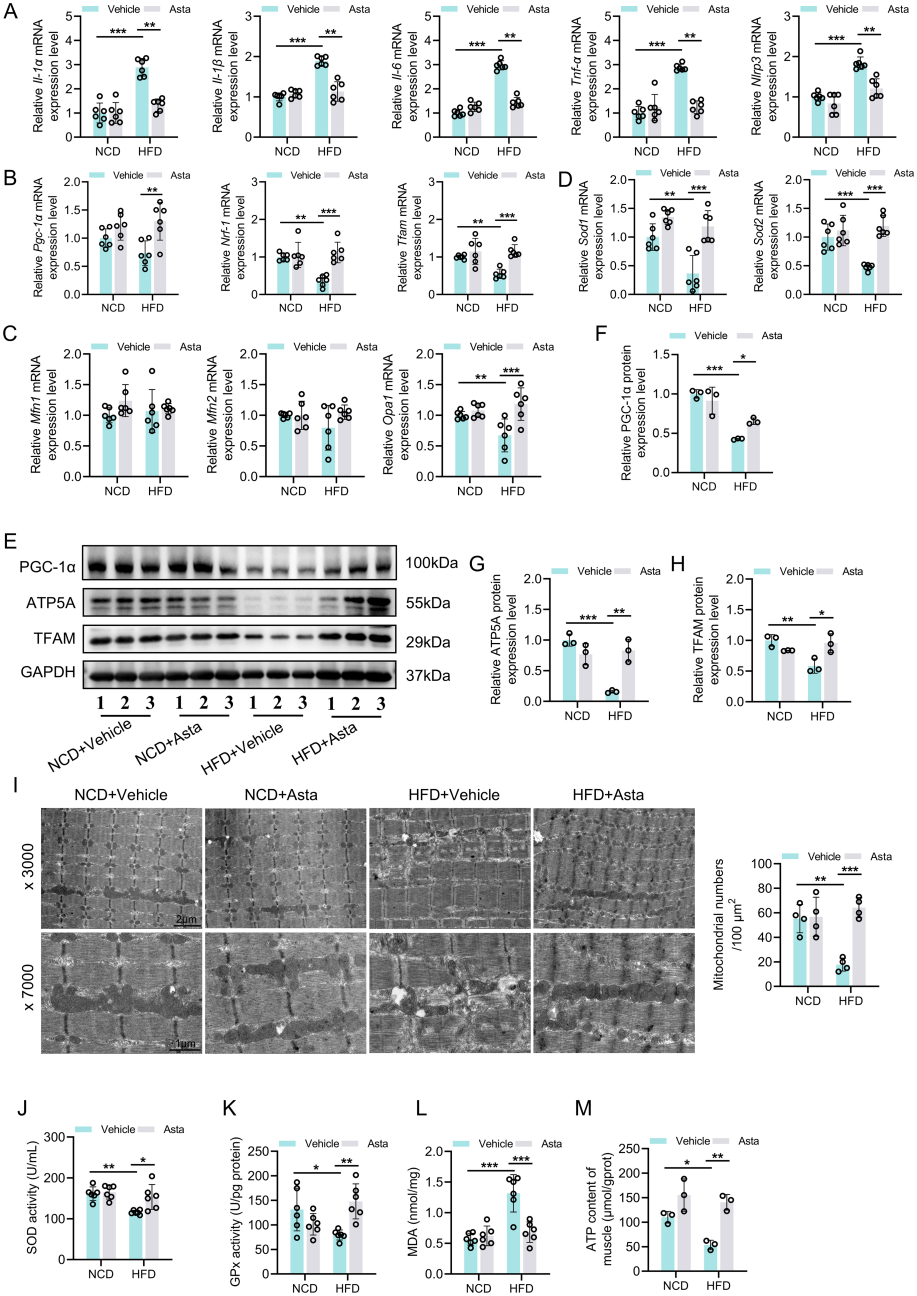
Astaxanthin (Asta) alleviates HFD-induced oxidative stress and mitochondrial biogenesis in skeletal muscles. **(A)** The expression levels of *Il-1α, Il-1β, Il-6, Tnf-α,* and *Nlrp3* genes (*n* = 6). **(B,C)** The expression levels of *Pgc-1α, Nrf1, Tfam, Mfn1, Mfn2,* and *Opa1* genes (*n* = 6). **(D)** The expression of *Sod1* and *Sod2* (*n* = 6). **(E–H)** Representative blot images and quantitative analysis of PGC-1α, TFAM, and ATP5A proteins (*n* = 3). **(I)** Representative transmission electron microscopy images of mitochondrial morphology (× 3,000, × 7,000) in skeletal muscle and quantitative analysis of mitochondrial numbers (*n* = 4). **(J,K)** SOD and GPx activity (*n* = 6). **(L)** Quantitative analysis of MDA levels (*n* = 6). **(M)** ATP content in skeletal muscles (*n* = 3). Data are shown as mean ± standard deviation (SD). Statistical analysis among four groups was performed using two-way ANOVA with a Sidak *post hoc test*. * *p* < 0.05, ** *p* < 0.01, and *** *p* < 0.001.

Mice fed HFD showed significant increased expression levels of inflammation-related genes in the skeletal muscle tissue compared to those fed NCD (Figure 2A). Additionally, expression levels of genes related to mitochondrial biogenesis, such as *Nrf1* and *Tfam*, was significantly reduced, while mitochondrial fusion-related genes remained unchanged in the HFD group, except for *Opa1* (Figures 2B,C). The genes related to anti-oxidative stress (*Sod1* and *Sod2*) were significantly downregulated in the HFD group compared to the NCD group (Figure 2D). Treatment with Asta for 12 weeks markedly inhibited expression of inflammation-related genes and promoted expression of *Pgc-1a, Nrf1, Tfam, Opa1, Sod1,* and *Sod2* genes (Figures 2A–D). At the protein levels, HFD-fed mice exhibited a marked reduction in PGC-1α, TFAM, and ATP5A expression levels in the skeletal muscles, accompanied by decreased mitochondrial numbers and disrupted mitochondrial structure (Figures 2E–I), reflecting impaired mitochondrial biogenesis. Asta treatment for 12 weeks generally restored expression levels of these proteins, increased mitochondrial numbers, and alleviated mitochondrial structure disruption in HFD-fed mice (Figures 2E–I). In HFD-fed mice, antioxidant enzyme activities, including SOD and GPx, were markedly reduced, while MDA levels, a marker of oxidative damage, were elevated (Figures 2J–L). Following 12 weeks of Asta treatment, the activities of SOD and GPx were significantly increased (Figures 2J,K), while MDA levels were markedly decreased (Figure 2L). The analysis of ATP content revealed that HFD feeding impaired ATP production in the skeletal muscles, reflecting disrupted mitochondrial energy metabolism. However, Asta treatment resulted in a restoration of ATP content compared to the HFD group (Figure 2M). Collectively, these findings suggest that Asta treatment enhances mitochondrial biogenesis and function in response to HFD-induced mitochondrial stress.

### Asta treatment reduces inflammation and lipid peroxidation in PA-exposed C2C12 cells *in vitro*

**Figure 3:**
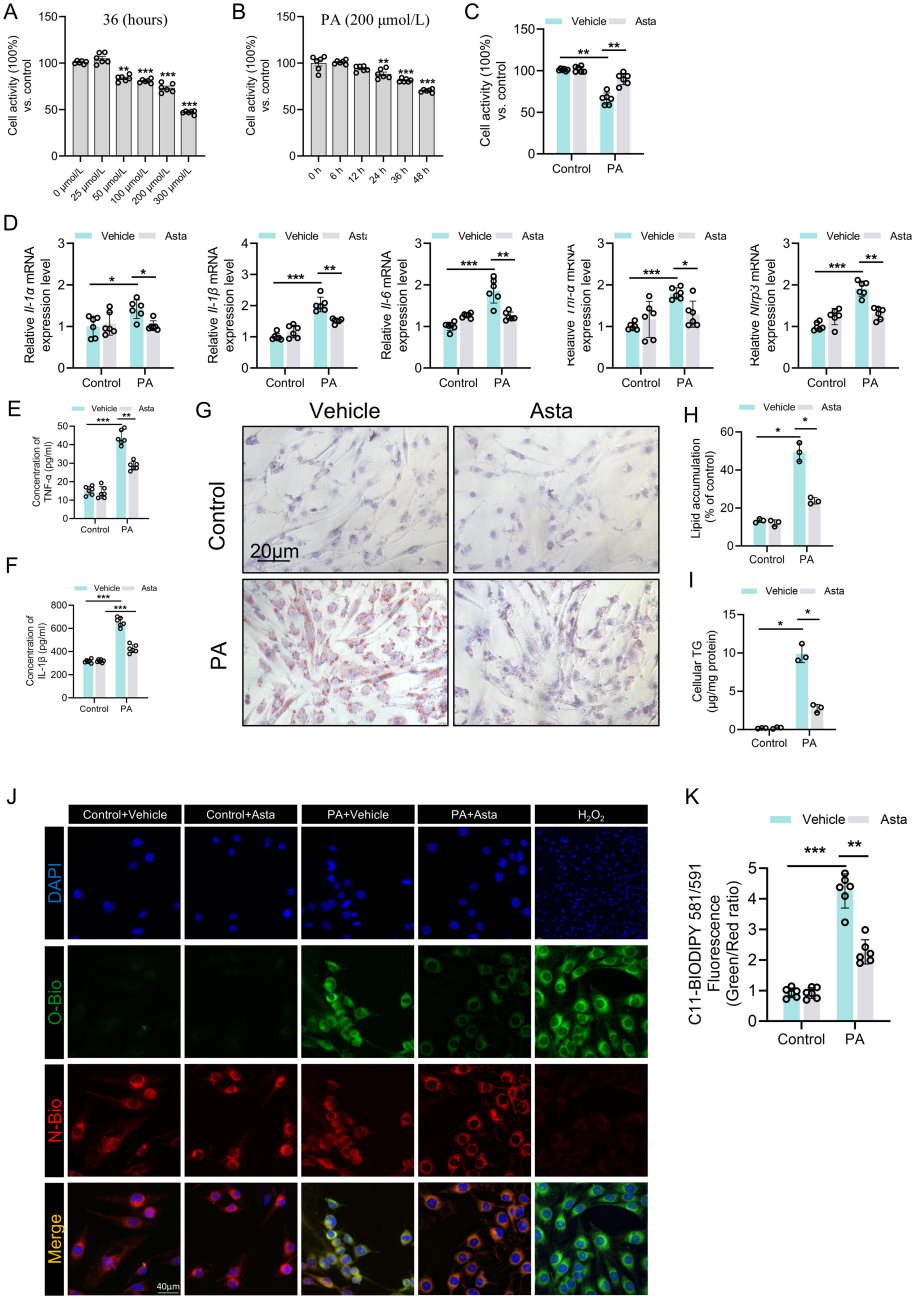
Astaxanthin (Asta) reduces inflammation and lipid peroxidation in palmitate acid (PA)-exposed C2C12 cells *in vitro*. **(A–C)** C2C12 cell activity (*n* = 6). **(D)** The expression levels of *Il-1α, Il-1β, Il-6, Tnf-α,* and *Nlrp3* genes (*n* = 6). **(E,F)** Quantitative analysis of intracellular TNF-α and IL-1β proteins in C2C12 cells (*n* = 6). **(G,H)** Oil Red O staining of C2C12 cells and quantitative analysis (*n* = 3). **(I)** C2C12 cells cellular triglycerides (TG) (*n* = 3). **(J)** The representative fluorescence images of C11 staining in C2C12 cells. **(K)** Quantitative analysis of fluorescence intensity ratio (Oxidized/ Non-oxidized). Data are shown as mean ± standard deviation (SD). Statistical analysis among four groups was performed using two-way ANOVA with a Sidak *post hoc test*. * *p* < 0.05, ** *p* < 0.01, and *** *p* < 0.001.

To determine the optimal induction condition, we first examined the effects of PA on C2C12 cell viability. In Figure 3A, increasing PA concentrations (0–300 μmol/L) for 36 h resulted in a dose-dependent decrease in cell viability, with 300 μmol/L causing approximately 50% loss. In Figure 3B, 200 μmol/L PA significantly reduced cell viability in a time-dependent manner, with greater reductions observed at 36 and 48 h. Accordingly, we selected 300 μmol/L PA for 36 h as the optimal induction condition. Asta treatment for 24 h after PA exposure significantly improved C2C12 cell viability (Figure 3C), demonstrating that Asta could mitigate PA-induced cell damage.

The results of qRT-PCR analysis showed that PA stimulation triggered pronounced inflammatory response in C2C12 cells, as evidenced by elevated mRNA expression levels of pro-inflammatory cytokines, including *Il-1α, Il-1β, Il-6, Tnf-α,* and *Nlrp3* genes (Figure 3D). The increased production of part cytokines was confirmed at the protein levels, with significant increases in intracellular TNF-α and IL-1β levels (Figures 3E,F). Lipid accumulation and intracellular TG content were significantly elevated after PA exposure, which were attenuated by Asta treatment (Figures 3G–I). Additionally, intracellular levels of C11, a marker of lipid peroxidation, were significantly elevated in PA-exposed C2C12 cells, which was reversed by Asta treatment (Figures 3J,K). Treatment with Asta for 24 h significantly reduced expression levels of inflammatory cytokine genes, including *Il-1α, Il-1β, Il-6, Tnf-α,* and *Nlrp3* (Figure 3D). Furthermore, Asta treatment lowered TNF-α and IL-1β protein levels, lipid accumulation, and lipid peroxidation in PA-exposed C2C12 cells (Figures 3E–I). Together, our findings indicate that Asta effectively reduces inflammatory and oxidative stress response induced by palmitate in C2C12 cells.

### Asta treatment mitigates mitochondrial damage and reduces oxidative stress in PA-exposed C2C12 cells *in vitro*

**Figure 4:**
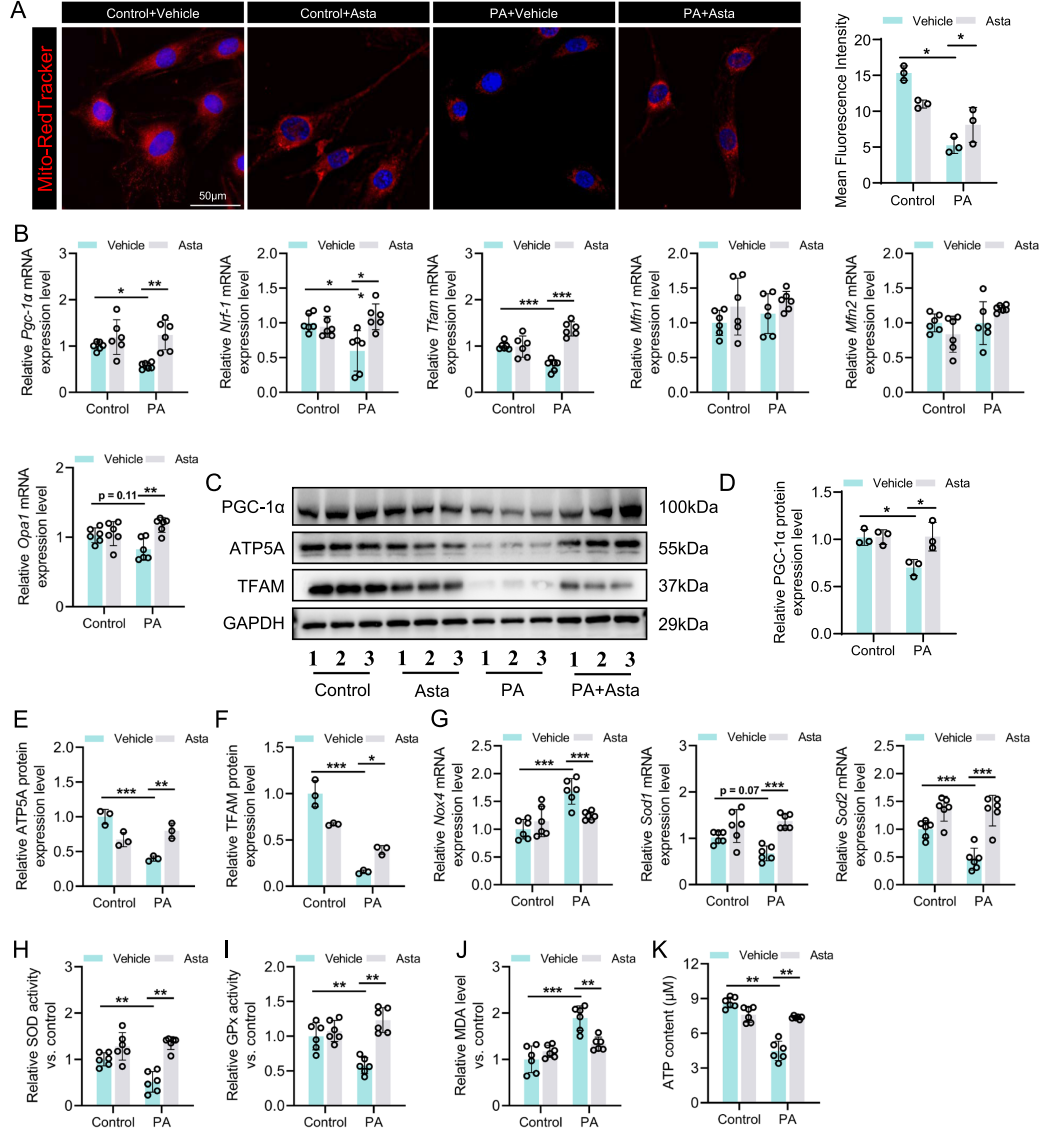
Astaxanthin (Asta) mitigates mitochondrial damage and reduces oxidative stress in palmitate (PA)-exposed C2C12 cells. **(A)** Mito-Tracker staining in C2C12 cells and quantitative analysis (*n* = 3). **(B)** The expression levels of *Pgc-1α, Nrf1, Tfam, Mfn1, Mfn2,* and *Opa1* genes (*n* = 6). **(C–F)** Representative blot images and quantitative analysis of PGC-1α, TFAM, and ATP5A proteins (*n* = 3). (G) The expression of *Sod1*, *Sod2,* and *Nox4* (*n* = 6). **(H,I)** SOD and GPx activity (*n* = 6). **(J)** Quantitative analysis of MDA levels (*n* = 6). **(K)** ATP content in skeletal muscle (*n* = 6). Data are shown as mean ± standard deviation (SD). Statistical analysis among four groups was performed using two-way ANOVA with a Sidak *post hoc test*. * *p* < 0.05, ** *p* < 0.01, and *** *p* < 0.001.

Subsequently, we continued to investigate the effects of Asta on mitochondria in cells in vitro. Our results showed that PA exposure led to significant mitochondrial structural damage in C2C12 cells, as evidenced by Mito-Tracker staining, with increased point and decreased filamentous mitochondria (Figure 4A). The downregulation of mitochondrial biogenesis genes such as *Pgc-1α, Nrf1,* and *Tfam*, not *Mfn1, Mfn2,* and *Opa1* (mitochondrial fusion), was observed in PA-exposed C2C12 cells (Figure 4B). Additionally, the results of western blotting analysis showed that PA exposure induced decreased expression of PGC-1α, TFAM, and ATP5A proteins in C2C12 cells (Figures 4C–F). Treatment with Asta for 24 h significantly mitigated mitochondrial defects in morphology (Figure 4A). The expression of mitochondrial biogenesis genes and the corresponding protein levels in C2C12 cells were restored by treatment with Asta (Figures 4B–F).

In terms of oxidative stress, PA exposure promoted the expression of oxidative stress-related gene (*Nox4*), but inhibited the expression of anti-oxidative stress genes (*Sod1 and Sod2*) in C2C12 cells (Figure 4G). These changes were accompanied by a decrease in activity of antioxidant enzymes (SOD and GPx) and an increase in oxidative production (MDA) (Figures 4H–J). Treatment with Asta reversed these effects, showing enhanced *Sod1* and *Sod2* expression, reduced *Nox4* expression, increased SOD and GPx activity, and reduced MDA levels (Figures 4G–J). Further analysis showed that PA exposure impaired ATP production in C2C12 cells (Figure 4K), which reflects impaired mitochondrial energy metabolism. Asta treatment restored ATP production to near control levels (Figure 4K), demonstrating that Asta is beneficial for protecting mitochondrial function and maintaining energy production in cells exposed to PA-induced stress.

## Discussion

In this study, we explored the protective effects of Asta on mitochondrial and skeletal muscles in the model of metabolic stress, focusing on HFD-fed mice *in vivo* and PA-exposed C2C12 cells *in vitro*. Our results show that Asta treatment suppresses inflammatory responses, attenuates lipid accumulation, and protects the structure and function of skeletal muscle. Mechanically, this role may be partly due to the protection of Asta on mitochondria, which is reflected in promoting mitochondrial biosynthesis, protecting mitochondrial structure and reducing oxidative stress. This implies that Asta has a promising therapeutic effect in protecting mitochondria and skeletal muscle damage.

One of the key findings of this study is the ability of Asta to promote mitochondrial biogenesis and protect mitochondrial damage, as evidenced by upregulated expression in PGC-1α, NRF1, and TFAM genes and proteins, as well as the changes in mitochondrial morphology and number. In fact, mitochondrial biogenesis is crucial for maintaining the number and function of mitochondria, which are the energy powerhouses of skeletal muscle cells (8, 9). Previous studies have extensively explored that HFD consumption and PA exposure resulted in impaired mitochondrial biogenesis and increased mitochondrial fragmentation, which reduce ATP production and compromise muscle performance (1, 5, 10, 18). Our results demonstrate that Asta treatment restores mitochondrial biogenesis markers, promotes increase in mitochondrial number, and mitigates mitochondrial structural damage, thereby improving its function. This is verified by elevated ATP production following Asta treatment in vivo and in vitro. Furthermore, the involvement of PGC-1α in regulating mitochondrial biogenesis may highlight the potential of Asta to activate critical pathways that safeguard mitochondrial function under metabolic stress (19).

While our findings indicate that astaxanthin improves mitochondrial morphology under metabolic stress, the underlying mechanisms related to mitochondrial dynamics remain unresolved. A modest upregulation of Opa1 mRNA was observed following Asta treatment, whereas other key fusion-related genes (Mfn1 and Mfn2) showed no significant changes, and no fission-related markers were assessed at either gene or protein levels. Definitive conclusions regarding the balance between mitochondrial fusion and fission cannot be drawn (20, 21). Additionally, it is important to recognize that mitochondrial dynamics are governed by a complex and often redundant regulatory network, and the alterations in fusion-related transcripts alone do not necessarily reflect alterations in fission activity. Moreover, existing evidence suggests that mitochondrial fission is not markedly affected in obesity without the presence of diabetes, where dysregulation becomes more apparent (22). Given these considerations, the observed improvements in mitochondrial morphology are more likely attributable to enhanced biogenesis and antioxidant capacity rather than direct modulation of the fusion-fission machinery (19). Therefore, further investigations using new ways targeting both fusion and fission pathways will be essential to clarify the specific effects of astaxanthin on mitochondrial quality control (23).

With mitochondrial function protected, significant reduction in oxidative stress levels is another crucial pathway for the protective effect of Asta on the skeletal muscles. Oxidative stress is typically driven by excessive ROS production. Previous studies indicated that HFD feeding and PA exposure increase ROS levels through mitochondrial disruption and lipid peroxidation, and the Nox4 pathway exacerbates oxidative stress (18, 24). In our study, Asta inhibits *Nox4* gene expression while enhancing the expression of antioxidant genes such as *Sod1* and *Sod2*, significantly reducing ROS production and the accumulation of lipid peroxidation markers (MDA). Additionally, Asta increases the activity of antioxidant enzyme, which contributes to further neutralizing ROS production (25). These pleiotropic antioxidant actions likely confer protection upon the mitochondrial membrane and attenuate ROS-mediated damage to mitochondrial DNA and proteins, safeguarding mitochondrial structural integrity and function. It is worth noting that the reduction in oxidative stress indirectly decreases inflammation (26). Chronic inflammation is implicated in skeletal muscle damage during metabolic stress, wherein excessive ROS production contributes not only to mitochondrial damage but also to the activation of inflammatory pathways and subsequent production of pro-inflammatory cytokines (26). In this study, Asta significantly downregulated the expression of pro-inflammatory genes and reduced the levels of inflammatory proteins, suggesting that its suppression of inflammatory signaling pathways may be mediated through the reduction of ROS production. Indeed, inhibiting inflammation response is crucial for preserving skeletal muscle function and minimizing muscle damage (27, 28). Asta treatment can break the vicious cycle between ROS and inflammation, providing further protection for the skeletal muscles.

The protective role of Asta in mitochondrial biogenesis, antioxidant action, and anti-inflammatory properties is ultimately exhibited in skeletal muscle structure and function. Our results show that HFD feeding causes muscle fiber thinning, loss of muscle mass, decreased locomotor activity and muscle strength; however, these damages are significantly improved by treatment with Asta. These findings suggest that Asta treatment not only protects mitochondria at the molecular and cellular levels, but also ameliorates HFD-induced skeletal muscle damage at the organ level.

Beyond Asta, several natural compounds have shown protective effects on skeletal muscle under metabolic stress. Resveratrol activates (sirtuin 1) SIRT1 and PGC-1α pathways, thereby promoting mitochondrial biogenesis and improving insulin sensitivity (29). Additionally, quercetin, curcumin, and epigallocatechin gallate also exert antioxidant and anti-inflammatory effects, attenuating muscle atrophy and mitochondrial dysfunction in the models of obesity or aging (30–32). Despite structural differences, these compounds share molecular mechanisms involving redox balance and mitochondrial homeostasis (32, 33). Compared to these agents, Asta possesses unique advantages, including a highly conjugated structure with antioxidant capacity and superior membrane permeability, allowing more efficient targeting of mitochondrial membranes (34). Although it shares pathways such as PGC-1*α* activation and antioxidant enzyme induction, Asta may provide stronger mitochondrial protection, particularly under lipid overload. However, further comparative studies are needed to fully assess its therapeutic potential relative to other nutraceuticals.

In conclusion, this study reveals that Asta treatment protects skeletal muscle against damage induced by metabolic stress through promoting mitochondrial biogenesis, protecting mitochondrial structure and function, and reducing oxidative stress and inflammation. The protective role of Asta has provided a foundation for its potential use in muscle-related metabolic diseases. In addition, it should be further clarified that the protective effects of astaxanthin on muscle are independent of its regulation of blood lipid and glucose levels. Therefore, future study ought to explore the clinical applications of Asta and elucidate its protective mechanisms in more complex physiological environments.

## Data Availability

The raw data supporting the conclusions of this article will be made available by the authors, without undue reservation.

## Glossary

Asta: Astaxanthin
HFD: High-fat diet
PA: Palmitate acid
NCD: Normal chow diet
CHOL: Cholesterol
TG: Triglycerides
LDL-C: Low-density lipoprotein cholesterol
HDL-C: High-density lipoprotein cholesterol
ATP: Adenosine triphosphate
MDA: Malondialdehyde
ROS: Reactive oxygen species
SOD: Superoxide dismutase
GPx: Glutathione peroxidase
PGC-1α: Peroxisome proliferator-activated receptor gamma coactivator 1-alpha
NRF1: Nuclear respiratory factor 1
TFAM: Transcription factor A, mitochondrial
IL-1β: Interleukin-1β
TNF-α: Tumor necrosis factor-alpha
Mfn1: Mitofusin 1
Mfn2: Mitofusin 2
Opa1: Optic atrophy 1
RT: Rotarod test
OFT: Open field test
ORO: Oil Red O
