# Supplementary material for: Astaxanthin alleviates oxidative stress and skeletal muscle damage by promoting mitochondrial biogenesis
Source: Front Vet Sci. 2025 Sep 2;12:1577408. doi: 10.3389/fvets.2025.1577408 (PMC12439717; doi:10.3389/fvets.2025.1577408)
Supplement: Supplementary file 1 [file Table_1.docx]

**Supplemental Table 1. Primers used in real-time polymerase chain reaction (RT-PCR).**

| **Gene** | **Species** | **Forward 5’ to 3’** | **Reverse 5’ to 3’** |
| --- | --- | --- | --- |
| ***Gapdh*** | Mouse | CAAGCTCATTTCCTGGTATGAC | AGGGAGATGCTCAGTGTTGG |
| ***Il-α*** | Mouse | AGCAGTCCCAACTAAGCAGTA | CAGCCAGTAGAGGATGCTGA |
| ***Il-β*** | Mouse | CACCCCCGGAACACCAAAG | CATACTCTTCCATTCGAGCGTAG |
| ***Il-6*** | Mouse | GCAGCATCACCTTCGCTTAGA | CAGATATTGGCATGGGAGCAAG |
| ***Nrf1*** | Mouse | CGTTGGATGAGTACACGACG | TTTCGCACCACATTCTCCAAA |
| ***Tfam*** | Mouse | GGATGATTCGGCTCAGGGAAA | AGCCATCTGCTCTTCCCAAG |
| ***Nox4*** | Mouse | GAAGGGGTTAAACACCTCTGC | ATGCTCTGCTTAAACACAATCCT |
| ***Nlpr3*** | Mouse | ATTACCCGCCCGAGAAAGG | TCGCAGCAAAGATCCACACAG |
| ***Opa1*** | Mouse | AGACTGTGTCAAAATCCTGCTC | AAGTGCCTGGAACACACGTA |
| ***Mfn1*** | Mouse | TTGGCAGGACAAGTAGTGGC | CTCTCTCTTTCGCACGGGTC |
| ***Mfn2*** | Mouse | AAACTGCTCAGGAATAAAGCTGG | AGCAGTTGGTTGTGTGACCA |
